# Follow-up investigation of asymptomatic COVID-19 cases at diagnosis in Busan, Korea

**DOI:** 10.4178/epih.e2020046

**Published:** 2020-06-23

**Authors:** Miyoung Lee, Youngduck Eun, Kyounghee Park, Jeonghun Heo, Hyunjin Son

**Affiliations:** 1 Busan Center for infectious Disease Control and Prevention, Pusan National University Hospital, Busan, Korea; 2 Epidemic Intelligence Officer of Busan Metropolitan City, Busan, Korea; 3 Epidemic Investigation Team of Busan Metropolitan City, Busan, Korea; 4 Division of Internal Medicine, Busan Medical Center, Busan, Korea

**Keywords:** COVID-19, SARS-CoV-2, Asymptomatic infection, Asymptomatic transmission

## Abstract

**OBJECTIVES:**

The objective of the study was to conduct a follow-up investigation of 10 asymptomatic patients at diagnosis among the 98 confirmed coronavirus disease 2019 (COVID-19) cases reported in Busan between February 21, 2020 and March 13, 2020 to determine whether asymptomatic infection and transmission during asymptomatic period are possible.

**METHODS:**

The study analyzed 10 asymptomatic, confirmed COVID-19 cases to determine whether asymptomatic infection is possible. We conducted in-depth interviews with patients and guardians; interviews with primary physicians; review of medical records and drug utilization review (DUR) reports; and base station-based location tracking.

**RESULTS:**

Among the 98, confirmed COVID-19 cases reported in Busan, the study analyzed 10 (10.2%) asymptomatic patients at diagnosis. The results confirmed that two (2.0%) patients reported to be asymptomatic during the initial epidemiological investigation, but turned symptomatic before diagnosis as per the in-depth interview results. Four cases (4.0%) of early detection led to confirmed diagnosis during the incubation period and presentation of symptoms after diagnosis. In addition, the remaining four patients (4.0%), having no subjective symptoms nor specific findings on chest radiography and computed tomography, remained asymptomatic until the isolation order was lifted. With regard to whether transmission during the asymptomatic period is possible, it was found that one out of 23 household contacts of the confirmed patients was identified as an additional confirmed case after coming in close contact with an index patient during the presymptomatic period.

**CONCLUSIONS:**

Among the 98 confirmed cases, asymptomatic infection was confirmed in four cases (4.0%). In addition, there was one additional confirmed case in which the patient was a family member who came in close contact with an index patient during the incubation period, thereby confirming that transmission during the asymptomatic period is possible. The possibility of transmission during the asymptomatic period has been confirmed; therefore, it is necessary to review the measures for expanding contact tracing that is currently being applied starting one day prior to the onset of symptoms.

## INTRODUCTION

Since the first confirmed case of coronavirus disease 2019 (COVID-19) in Busan on February 21, 2020, a total of 98 confirmed cases have been reported over a 22-day period, up to March 13, 2020. Epidemiological investigation conducted immediately upon reporting found that 10 patients (10.2%) were confirmed to have COVID-19 infection while being asymptomatic. COVID-19 being a novel infectious disease, characteristics of the virus and epidemiological characteristics of the disease are being newly identified with an increase in the number of patients, while there is much confusion due to various issues including possible asymptomatic transmission, aerosol transmission, and possibility of reinfection. In particular, much interest is being focused on asymptomatic infection since it is an issue that directly impacts the infectious disease crisis response, such as estimation of incubation and transmission periods during the current situation of declaration of a pandemic.

On January 29, 2020, the World Health Organization (WHO) issued guidelines to investigate anyone who has been in close contact with a confirmed patient, starting from at least one day prior to the onset of perceived symptoms, in consideration of the risk of asymptomatic transmission [1], thereby suggesting the possibility of asymptomatic transmission. Subsequently on February 21, 2020, a Chinese research team published an article titled “Presumed asymptomatic carrier transmission of COVID-19” [2] in the *Journal of the American Medical Association* (JAMA), which also recognized asymptomatic infection and transmission. In light of this information, the Korea Centers for Disease Control and Prevention (KCDC) changed its position from completely dismissing the possibility of asymptomatic transmission to recognizing the possibility of asymptomatic infection and transmission, and advocating the need to strengthen preemptive and aggressive disease control measures [3].

Accordingly, the present study conducted in-depth reinvestigation and medical records review of 10 asymptomatic cases reported in Busan to analyze the cases with confirmed COVID-19 infection prior to the onset of patient-perceived symptoms in an effort to find the answers to the questions “Is asymptomatic infection possible?” and “Is person-to-person transmission possible in the asymptomatic state?”

## MATERIALS AND METHODS

Ten asymptomatic, confirmed COVID-19 cases reported in Busan between February 21, 2020 and March 13, 2020 were investigated to find if asymptomatic infection transmission is possible. Data were collected through in-depth investigation of patients and their family members, interviews with primary physicians, and review of medical records. Follow-up investigation was conducted on the status and date of symptom onset until complete recovery, based on the comparison of statements given by patients and family members, opinions of primary physicians on patient condition, and test results such as real-time reverse transcription polymerase chain reaction (PCR), chest radiography, and computed tomography (CT) scan results. Possibility of transmission during the asymptomatic period was studied through the review of epidemiological investigation result of confirmed case among 23 close contacts of 10 asymptomatic cases.

## RESULTS

To check whether asymptomatic infection is possible, the present study analyzed 10 (10.2%) confirmed COIVD-19 cases who were in an asymptomatic state, among the 98 confirmed cases reported in Busan between February 21, 2020 and March 13, 2020.

**Figure 1.**
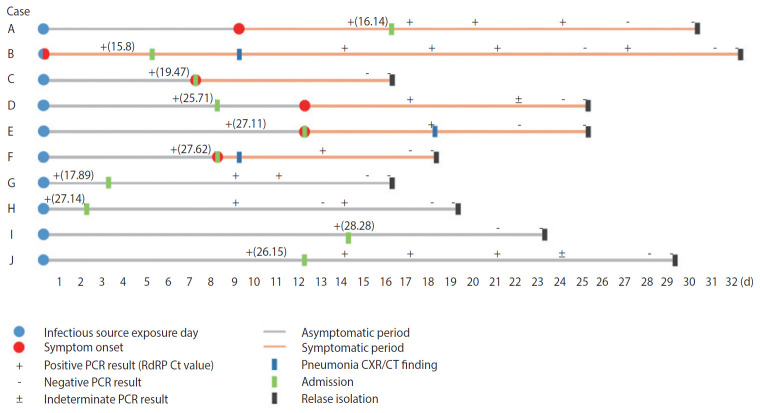
Timeline of ten asymptomatic coronavirus disease 2019 (COVID-19) confirmed cases. PCR, real-time reverse transcription polymerase chain reaction; RdRP, RNA-dependent RNA polymerase; Ct, cycle threshold; CXR, chest X-ray; CT, computed tomography.

The results confirmed that two cases (2.0%) that reported to be asymptomatic in the initial epidemiological investigation changed their statement to being symptomatic; four cases (4.1%) with confirmed diagnosis during the incubation period presented with symptoms after the diagnosis; and four cases (4.1%) were asymptomatic until the isolation order was lifted (Figure 1).

The statements given by the two patients who changed their statement (Patients A and B) and their family members during the epidemiological investigation after reporting were inconsistent, and were revised at a later time. The parents of Patient A stated that the patient showed symptoms of a cold prior to being diagnosed with COVID-19. However, the patient initially reported no symptoms, but subsequently admitted to having symptoms prior to confirmation of the diagnosis during the interview with the primary physician after hospitalization. Patient B had difficulty in responding to the telephone survey due to old age, and as a result, the spouse provided the responses instead. However, the patient admitted to having symptoms prior to confirmed diagnosis during the face-to-face interview with the primary physician after being admitted to the isolation unit.

All four cases with onset of symptoms after confirmed diagnosis (Patients C, D, E, and F) belonged to the group of outbreak cases, and as a result, the patients were subjected to total testing regardless of symptom presentation. These patients experienced onset of symptoms at 0, 1, 2, and 5 days after the diagnosis, and pneumonia was found on chest radiography and CT in two patients (Patients E and F).

The findings in the four cases that were asymptomatic until lifting of the isolation order (Patients G, H, I, and J) were as follows. Patient J complained of headaches, but this was a usual symptom for the Patient J, and the patient did not feel any temporal pattern change, such as the worsening of such symptoms. In addition, the patient did not experience the onset of any other symptoms until discharge. Patient H was a 5-year-old boy who was hospitalized in the asymptomatic state, who did not present with any specific symptoms and received no medication until the lifting of the isolation order. Patients G and I also remained asymptomatic until complete recovery, but these two patients had received drug therapy (hydroxychloroquine) starting from the day after admission. And they went on drug for 13 days, 9 days. All four asymptomatic patients had no specific findings on chest radiography or CT (Table 1).

Excluding the two patients who changed their statements about the onset of symptoms during the in-depth interviews, 71 people who came in contact with the other eight patients were identified. Of these 71 people, 23 who came in close contact (household contact) were investigated to identify asymptomatic transmission. The contact period of index patient was defined as the period from the last day of exposure until isolation date. Based on this definition, the mean contact period was calculated to be 7.7 days. Patient I had the longest contact period of 14 days with close contacts. However, considering that Patient I was complying with the self-quarantine order, Patient E, who was not in self-quarantine, may have had the longest contact period of 12 days. Among a total of 19 people who had come in contact with Patient E, there were three household contacts, and there were no additional new cases except Patient K. The exposure period among other index patients besides Patient E, ranged between as short as 2 days (Patient H) to as long as 8 days (Patients D and F), which were all shorter than that of Patient E. Moreover, considering that Patient E, who is the index patient of Patient K, stated that he mostly stayed at home due to school vacation, the actual intensity of contact may have been much higher than that of other index patients and their contacts.

One additional case was identified by performing pre-emptive PCR test, together with self-quarantine for household contacts. Pre-emptive PCR test refers to the PCR test performed immediately upon identification of household contacts. It is a part of the aggressive response measures established for the early detection of patients and blocking further transmission due to COVID-19 characteristically having high transmissivity in the early stage of infection [4]. A recent study indicated that the possibility of asymptomatic transmission cannot be dismissed [3], and a high incidence of additional cases among household contacts of confirmed patients was identified by monitoring the additional cases that occurred in Busan. COVID-19 response guidelines 7-4th edition [5], recently revised by KCDC, requires two rounds of testing immediately upon discovery for household contacts, before lifting of the quarantine order.

**Figure 2.**
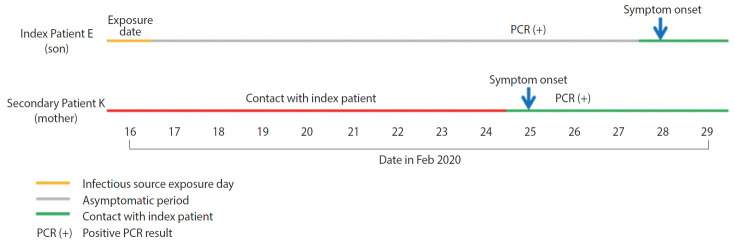
Timeline of exposure to asymptomatic coronavirus disease 2019 (COVID-19) index patient. PCR, real-time reverse transcription polymerase chain reaction.

The additional patient (Patient K) was the mother of index Patient E, who was diagnosed while in self-quarantine. The onset of symptoms developed earlier in Patient K than Patient E, and location tracking based on the mobile base stations, review of drug utilization review (DUR) reports, and in-depth interviews were conducted to identify the temporality of transmission. Review of DUR reports showed that Patient K had not visited a medical institution or pharmacy within 14 days prior to the onset of symptoms, while base station-based location tracking indicated that she had visited Seoul for four days (February 12 to February 15, 2020). However, there were no specific findings related to possible transmission of COVID-19 on this travel (Figure 2).

## DISCUSSION

Among the 10 asymptomatic, confirmed COVID-19 cases, two were symptomatic prior to the diagnosis, but were recorded as asymptomatic in the initial epidemiological investigation, only to have their statement changed at a later time. To prevent this from happening, a more thorough approach in the epidemiological investigation process is necessary. Most epidemiological investigations are carried out within a few hours of receiving a confirmatory diagnosis, and as a result, the patient may be fearful just from the fact that he or she has been diagnosed. In addition, epidemiological investigations have strong traits of investigating the movement pattern of patients and identifying people who have come in contact, rather than concern about the health of the patient, and as a result, the fear and anxiety experienced by the patient may be heightened even further. This could act as one of the elements that cause the patient to unwittingly hide the truth or give inaccurate information. Therefore, it is necessary to conduct the epidemiological investigation after the patient has become stable, by providing enough emotional support.

Four patients, who were found to be asymptomatic in the initial epidemiological investigation after diagnosis but experienced onset of symptoms after hospitalization were diagnosed during the incubation period through early testing. These patients were tested as a part of primary testing of household contacts of confirmed patients or full contact tracing and testing, meaning, the tests were performed regardless of the presence of symptoms. Such a field testing performance is slightly different from the COVID-19 response guidelines 7-3th edition (for public health units) [6] put forth by KCDC, which stipulates that when symptoms appear during self-quarantine, primary test should be performed. Considering the high secondary attack rate and asymptomatic transmission among household contacts, testing guidelines of household contacts need to be modified to fit the actual testing performance.

The remaining four cases were of asymptomatic infection, of which, two patients (Patients G and I) received drug therapy starting from the day after hospitalization. Although it may be necessary to check the possibility of expression of symptoms being inhibited by the drug, they achieved complete recovery and remained asymptomatic until isolation was lifted. These four patients not only had no self-perceived subjective symptoms, but also showed no specific test findings or clinical outcomes.

COVID-19 is a novel infectious disease, and as a result, there are not many published study results on asymptomatic infection. A recently reported outbreak at the Guro Call Center in Seoul showed an asymptomatic infection rate of 8.2% [7], whereas asymptomatic infection was found in only 4 out of 98 cases (4.0%) in Busan, in the present study. Although the rate was slightly lower, both results confirmed that asymptomatic infection is possible.

Investigation of close contacts of asymptomatic, confirmed patients identified one additional patient (1.0%; Patient K). It appeared that Patient K was infected by Patient E, a household contact who was an asymptomatic, confirmed patient. Investigation of the outbreak at Guro Call Center in Seoul with 16 asymptomatic patients at diagnosis for determination of transmission during the asymptomatic incubation period did not yield any evidence of transmission [7]. However, the present study identified one case of presymptomatic transmission.

Patient K, who was a close contact of a confirmed patient and subsequently confirmed to be infected with COVID-19, had a family member (Patient E) who was diagnosed positively and had onset of symptoms earlier. Therefore, a multi-faceted investigation was conducted to determine the temporality of infection between these two patients. The results showed that a clear source of infection could not be found in Patient K except for only having contact with Patient E; no history of overseas travel, visiting a COVID-19 outbreak location, nor contact with other confirmed patients was elicited. On the other hand, the source of infection was very clear in Patient E. Therefore, it is valid to believe that Patient K was infected by Patient E. To identify the source of infection in Patient K, DUR and location tracking for February 1, 2020 to February 26, 2020 were checked. During this period, Patient K had visited Busan ○○ Church (church that Patient K usually went to) and Seoul (February 12 to February 15, 2020). However, there were no other reported cases from Busan ○○ Church other than Patient K, and location tracking information confirmed that Patient K went from Kimpo Airport to S Hospital and stayed at the hospital during the visit to Seoul, without visiting any other places. Therefore, there were no scenarios hinting at a possible source of infection with the visit to Seoul. On the other hand, Patient E was confirmed to have attended a church retreat where a group outbreak occurred in Busan, whereby a definite route of exposure to the source of infection was identified. Moreover, Patient E returned home from the retreat during school vacation, and during the in-depth interview reported of having stayed home for a long time. Therefore, it is suspected that close contact occurred continuously during this period. Considering these situations, it is believed that even though the onset of symptoms may have occurred earlier in Patient K, Patient E actually transmitted COVID-19 to Patient K during the presymptomatic period. Such findings were in the same context as a German study which found and reported transmission during the asymptomatic incubation period between coworkers [8].

The WHO [9] and China [10] are conducting contact tracing starting from two days prior to onset of symptoms in confirmed patients in consideration of possible asymptomatic transmission, whereas COVID-19 response guidelines 7-3th edition (for public health units) [6] of Korea requires contact tracing starting from 1 day prior to onset of symptoms. It is a known fact that COVID-19 has strong transmissivity during the early stage of infection, and it is necessary for Korea to re-evaluate the transmissible period in consideration of asymptomatic transmission, by expanding contact tracing to respond to such infectivity and block further transmission.

The investigation identified a total of four cases (4.1%) of asymptomatic infection among 98 confirmed cases reported in Busan. Among those four cases, two (2.0%) may need further review based on the possibility of the drug therapy they received upon hospitalization, which could have inhibited the onset of symptoms. Even so, the other two (2.0%) patients who remained asymptomatic until the isolation order was lifted confirmed the possibility of asymptomatic infection.

A family member of a confirmed patient who came in close contact during the presymptomatic period was identified as one additional patient. This implies that transmission is also possible during the presymptomatic period. However, the investigation had a small sample size of only 10 cases. Therefore, additional studies will be needed in the future; and more extensive investigation through immunological tests will be needed to obtain clearer and more scientific results on the asymptomatic infection rate.

## Figures and Tables

**Table 1.** Follow-up investigation results of ten asymptomatic confirmed coronavirus disease 2019 (COVID-19) cases in Busan

Case	Age (yr)	Sex	Underlying diseases	Exposure date	Source of infection	Diagnosis date	Admission period	Symptom onset date	Diagnosis to symptom onset period (d)	Symptoms	Pneumonia (date)	Medication (hydroxychloroquine or Kaletra)
A	21	M	None	Feb 8	Imported from another country	Feb 23	Feb 24-Mar 10	Feb 17	Exception	Cough, sputum, diarrhea	None	Taken
B	79	M	None	Feb 21	Church A	Feb 25	Feb 26-Mar 25	Feb 21	Exception	Headache, weakness	Detected (Mar 1)	Taken
C	30	F	None	Feb 17	Church A	Feb 23	Feb 24-Mar 5	Feb 24	1	Sore throat	None	None
D	21	F	None	Feb 16	Church A	Feb 23	Feb 24-Mar 14	Feb 28	5	Chills, headache, myalgia	None	Taken
E	25	M	Rhinitis	Feb 16	Church A	Feb 26	Feb 28-Mar 14	Feb 28	2	Cough, rhinorrhea, stuffy nose	Detected (Mar 5)	Taken
F	7	M	None	Feb 21	Contacts	Feb 29	Feb 29-Mar 11	Feb 29	Same day	Cough	Detected (Mar 1)	None
G	48	F	Rhinitis	Feb 23	Contacts (household)	Feb 24	Feb 26-Mar 11	Asymptomatic	None	None	None	Taken
H	5	M	None	Feb 29	Contacts (household)	Mar 1	Mar 02-Mar 20	Asymptomatic	None	None	None	None
I	25	M	None	Feb 18	Shincheonji	Mar 4	Mar 03-Mar 13	Asymptomatic	None	None	None	Taken
J	38	F	None	Feb 24	Contacts (household)	Mar 6	Mar 07-Mar 24	Asymptomatic	None	None	None	None

M, male; F, female.

## SUPPLEMENTARY MATERIALS

Korean version is available at http://www.e-epih.org/.
