# Supplementary material for: Follow-up investigation of asymptomatic COVID-19 cases at diagnosis in Busan, Korea
Source: Epidemiol Health. 2020 Jun 23;42:e2020046. doi: 10.4178/epih.e2020046 (PMC7644929; doi:10.4178/epih.e2020046)
Supplement: Supplementary file 1 [file epih-42-e2020046-suppl.doc]

**부산광역시 코로나바이러스감염증-19 무증상 확진자 추적조사**

**Follow up investigation of asymptomatic COVID-19 cases at diagnosis in Busan, South Korea**

저자: 이미영^1)2)^, 은영덕^1)2)^, 박경희^1)3)^, 허정훈^4)^, 손현진^1)2)^

소속: 1) 부산대학교병원 부산광역시 감염병관리지원단 2) 부산광역시 역학조사관 3) 부산광역시역학조사반 4) 부산광역시 부산의료원 내과

저자(영문): Miyoung Lee^1)2)^, Youngduck Eun^1)2)^, Kyounghee Park^1)3)^, Jeonghun Heo^4)^, Hyunjin Son^1)2)^

소속(영문): 1) Busan Center for infectious Disease Control and Prevention, Busan National University Hospital, Busan, Korea 2) Epidemic Intelligence Officer of Busan Metropolitan City, Busan, Korea 3) Epidemic investigation team of Busan Metropolitan City, Busan, Korea 4) Division of Internal Medicine, Busan medical Center, Busan, Korea

Correspondence: Hyunjin Son, 14F, 1001 Jungang-daero, Yeonje-gu, Busan, Korea 47545, hjson78@gmail.com

**ORCID Id**

Hyunjin Son (https://orcid.org/0000-0002-6571-5109)

**초록 Abstract**

**목적(Objectives):** 2020년 2월 21일부터 3월 13일까지 부산시에 신고된 98명의 코로나바이러스감염증-19 환자 중 진단 당시 무증상이었던 10명의 환자를 추적 조사하여 무증상 감염이 가능한가, 무증상기에도 전파가 가능한가에 대해 확인해 보고자 하였다.

**방법(Methods):** 무증상 감염이 가능한가를 보기 위해 무증상 상태에서 확진된 10명의 사례를 분석하고 환자 및 보호자 대상 심층 면담, 주치의 면담, 의무기록 및 DUR(Drug Utilization Review) 열람, 기지국 기반 위치추적 등을 시행하였다.

**결과(Results):** 부산시 확진 환자 98명 중 무증상 상태에서 코로나바이러스감염증-19에 확진된 10명(10.2%)의 사례를 분석한 결과 이 중 2명(2.0%)은 초기 역학조사에서 무증상이라고 하였다가 심층면담을 통해 진단 전 이미 증상을 가지고 있었던 것으로 변경된 경우였고, 4명(4.0%)은 조기 발견된 사례로 잠복기에 진단 후 증상이 발현된 경우였다. 그러나 나머지 4명(4.0%)은 환자 본인이 느끼는 주관적 증상이 없었고 Chest x-ray 및 CT 촬영 결과에서도 특이점을 발견할 수 없었으며 격리 해제 시까지 무증상이었다. 무증상기에 전파가 가능한가에 대해서는 확진 환자의 가족 등 밀접접촉자 23명 중 지표환자의 무증상기에 밀접 접촉한 1명이 추가 환자로 확진되었다.

**결론(Conclusions):** 98명의 확진 환자 중 총 4명(4.0%)의 무증상 감염 환자를 확인하였고 또한 환자의 무증상기에 밀접 접촉한 가족 중 1명이 확진되어 무증상기에 전파가 가능함을 확인할 수 있었다. 무증상기 전파가 확인됨에 따라 현재 증상발생 1일 전부터 시행하고 있는 환자의 접촉자 조사 범위를 확대 시행하는 방안에 대한 검토가 요구된다.

* 중심단어(Key words) – COVID-19;SARS-CoV-2;asymptomatic infection, asymptomatic transmission

**서론 Introduction**

2020년 2월 21일 부산에 코로나바이러스감염증-19 첫 확진환자가 발생한 후 3월 13일까지 22일 동안 총 98명의 환자가 신고되었으며, 신고 즉시 시행한 역학조사 결과 10명(10.2%)이 무증상 상태에서 확진된 것으로 보고되었다. 코로나바이러스감염증-19는 신종 감염병인만큼 환자가 증가하면서 바이러스의 특징 또는 역학적 특성들이 발견되고 있기도 하고 무증상 상태에서의 전파 가능성, 에어로졸을 통한 전파, 재감염 가능성 등 많은 이슈들로 인해 혼란을 겪고 있다. 특히 무증상 감염이 가능할 것인가의 문제는 Pandemic이 선언된 현 상황에서 잠복기와 전파기간의 추정 등 감염병 위기 대응에 직접적인 영향을 미치는 이슈로서 관심이 집중되고 있다.

세계보건기구(WHO)는 1월 29일 무증상 전파 위험을 고려하여 환자의 첫 자각증상 발생 하루 전부터 밀접 접촉자를 조사하라는 지침[1]을 내림으로써 무증상 전파의 가능성을 시사했고, 연이어 2월 21일 중국 연구진이 미국의학협회지(JAMA)에 게재한 '코로나19 무증상 보균자 전파 추정’[2] 이라는 논문도 무증상 감염과 전파를 인정하고 있다. 이에 따라 한국의 질병관리본부도 초기에 무증상 전파 가능성을 전면 부인하던 것에서 무증상감염과 무증상 전파 가능성을 인정하고 선제적이고 과감한 방역대책을 강화할 필요가 있다고 입장을 변경하였다.[3]

이에 본 연구는 부산시에 보고된 10건의 무증상 사례에 대해 심층 재조사 및 의무기록 조사를 실시하는 등 환자의 자각증상 발생 전 코로나바이러스감염증-19로 확진되는 사례들에 대한 분석을 통해 무증상 감염은 가능한가?, 무증상 감염 상태에서의 사람 간 전파는 가능한가? 에 대한 답을 찾아보고자 하였다.

**대상 및 방법 Materials and Methods**

무증상감염이 가능할 것인가에 대한 조사는 2020년 2월 21일부터 3월 13일까지의 부산광역시 코로나바이러스감염증-19 신고사례 중 무증상 확진 사례 10건에 대해 환자 및 가족에 대한 심층 조사 실시, 주치의 면담, 의무기록 열람 등의 방법을 사용하여 자료를 수집하였고, 환자 및 가족의 진술 대조, 임상증상 등 환자 상태에 대한 주치의 의견과 real time RT-PCR, Chest x-ray 및 CT 촬영 등 검사결과를 근거로 완치될 때까지의 증상발생 여부 및 증상발생일을 추적 조사하였다. 10명의 무증상 환자의 밀접접촉자 23명 중 확진된 환자에 대한 역학조사 결과 검토를 통해 무증상기 전파 가능성을 확인하였다.

**결과 Results**

본 조사는 먼저 무증상 감염 가능성을 확인하기 위해 2020년 2월 21일-3월 13일까지 부산시에서 확진된 환자 98명 중 무증상 상태에서 코로나바이러스감염증-19에 확진된 10명(10.2%)의 사례를 분석하였다.

이들에 대한 심층조사를 시행한 결과 초기 역학조사에서 무증상이라고 하였다가 후에 증상이 있었다고 진술이 변경된 환자가 2명(2.0%), 진단 후 증상이 발현된 환자가 4명(4.0%)이었고, 4명(4.0%)은 격리 해제 시까지 무증상이었다.(Figure 1)

진술이 변경된 2명(A, B)의 환자는 모두 신고 후 역학조사에서 본인과 가족의 진술이 불일치했다가 번복된 경우이다. A의 경우 부모는 환자가 확진 이전부터 감기증상이 있었다고 대답하였는데 본인은 증상이 없다고 하였다가 입원 후 주치의와의 면담에서 확진 전 증상이 있었다고 대답하였다. B의 경우는 고령자로서 유선조사에 어려움이 있어 배우자가 대신 면담에 응한 경우로 격리병상 입원 후 환자 본인과 주치의와의 면담 시 확진 전 증상이 있었다고 진술하였다.

확진 이후 증상이 발생한 4명(C, D, E, F)의 경우 모두 집단 발생 사례에 속하여 증상 발현과 관계없이 전수검사를 받은 경우들이다. 이들은 각각 진단일로부터 0일, 1일, 2일, 5일 후에 증상이 나타났으며 이 중 2명(E, F)은 Chest X-ray나 CT상 폐렴이 확인되었다.

격리해제 시까지 무증상이였던 4명(G, H, I, J)의 조사결과는 다음과 같다. 환자 J는 두통을 호소하였으나 이는 평소에도 항상 있었던 증상이며 증상이 심해지는 등 평소와 다른 어떤 변화도 느끼지 못했다고 진술하였고, 퇴원 시까지 그 외에 다른 증상 발현은 없었다. H는 5세 남아로 무증상 상태에서 입원하였고 격리해제 시까지 특이적인 증상이나 투약 내역은 없었다. G와 I도 완치 시까지 무증상이었는데 이 두 환자의 경우에는 입원한 다음 날부터 치료제(Hydroxychloroquine)가 투여되었고 각각 13일, 9일간 복용하였다. 무증상이었던 4명의 경우 Chest X-ray나 CT상에서도 특이소견이 없었다. (Table)

무증상 감염 상태에서의 전파를 파악하기 위해 심층면담에서 증상발생 여부가 변경되었던 2명을 제외한 환자 8명의 접촉자 총 71명 중 밀접 접촉자(동거가족) 23명을 조사하였다. 지표환자의 접촉기간은 노출 마지막 날부터 격리일까지로 정의했다. 이를 기준으로 기간을 산출했을 때 평균 접촉기간은 7.7일이었다. 환자 I는 밀접접촉자와 접촉기간이 14일로 가장 길었지만 환자 I가 자가격리 상태로 수칙을 준수하고 있었던 점을 고려하면 자가격리 상태가 아닌 E의 접촉기간이 12일로 가장 길었다. E의 접촉자 총 19명 중 가족 접촉자는 3명이었으며 K 이외에 추가 환자 발생은 없었다. 또한 E를 제외한 다른 지표환자들의 접촉기간을 보면 짧게는 2일(H)부터 길게는 8일(D, F)로 E보다는 짧았으며 더욱이 K의 지표환자인 E가 방학기간 중으로 주로 집에 머물렀다는 진술을 고려하면 다른 지표환자와 접촉자 간의 접촉보다 실제 접촉의 강도가 훨씬 더 높았을 것으로 추정할 수 있다.

가족 접촉자에 대해서는 자가격리 조치와 동시에 선제적인 PCR 검사를 시행하여 1명의 추가 환자를 확인하였다. 선제적인 PCR 검사란 가족 등 밀접접촉자 발견 즉시 시행하는 1차 PCR 검사를 말한다. 이는 코로나바이러스감염증-19 특성상 감염 초기에 전파력이 크며[4] 무증상감염의 전파 가능성[3]을 배제할 수 없다는 최근의 연구결과들과 그간 부산시에서 발생한 추가 환자에 대한 모니터링 결과 확진 환자의 가족에서 추가 환자 발생이 많았으므로 조기에 환자를 발견하고 전파를 차단하기 위해 마련한 부산시의 적극적인 대응방안의 일환이다. 최근 질병관리본부도 개정된 코로나바이러스감염증-19 대응지침 7-4판 [5]에서 가족 접촉자에 대해 발견 즉시와 격리해제 전 2회의 검사를 시행하도록 하고 있다.

추가 환자(이하 K)는 지표환자 E의 어머니로 자가격리 상태에서 확진되었다. K가 E보다 증상발생이 선행하였으므로 E와 K의 전파 선후관계를 확인하기 위해 기지국 기반 위치추적, DUR 확인 등과 심층면담을 실시하였다. DUR 확인결과 K는 증상발생 14일 전 의료기관이나 약국 방문은 없었으며 기지국 기반 위치추적에서는 2월 12일부터 15일까지 4일간 서울을 방문했던 것을 확인할 수 있었으나 코로나바이러스감염증-19의 전파와 관련된 특이점은 찾을 수 없었다.(Figure 2)

**고찰 Discussion**

무증상으로 확진된 10명 중 2명은 진단 전 증상이 있었으나 진단 후 초기 역학조사에서 무증상으로 조사되었다가 진술이 변경된 경우에 해당한다. 이와 같은 사례가 발생하지 않기 위해서는 역학조사 과정에서의 보다 섬세한 접근이 필요할 것이다. 대개 역학조사는 환자가 확진 판정을 받고 수 시간 이내에 이루어지므로 이때 환자는 진단되었다는 사실만으로도 공포감을 가지게 된다. 더욱이 역학조사가 환자의 건강에 대한 배려보다는 환자의 이동 동선을 파악하고 접촉자를 확인하기 위한 조사의 성격이 강할 때 환자가 느끼는 두려움이나 불안감은 더 커질 수 있다. 이는 자신도 모르게 사실을 가리거나 부정확하게 만드는 요소 중 하나로 작용할 수 있으므로 충분한 정서적 지지와 함께 환자가 안정된 상태에서의 역학조사의 시행이 요구된다.

진단 후 역학조사에서 무증상자로 조사되었으나 입원 후 증상이 나타난 4명은 조기검사에 의해 잠복기에 진단된 사례였다. 이들의 검사동기는 확진 환자의 밀접 접촉자에 대해 시행하는 1차 검사, 또는 집단발생 사례에 대해 시행된 접촉자 전수검사가 계기가 되었으므로 증상발생과 상관없이 검사가 시행되었다. 현장의 이러한 검사체계는 자가격리 중 증상이 나타나면 1차 검사를 하고, 음성인 경우 자가격리를 유지하다 해제 전 한 번 더 검사를 실시하도록 규정한 질병관리본부의 「코로나바이러스감염증-19 대응지침 7-3판(지자체용)」[6]과는 다소 차이가 있다. 가족접촉자의 높은 2차 발병률과 무증상 전파를 고려하여, 검사 지침의 개정을 검토해 보아야 한다.

나머지 4명은 무증상 감염 사례로 그 중 2명(G, I)은 입원 다음날부터 치료제가 투여되었으므로 약제의 효과로 인해 증상 발현이 억제되었을 가능성에 대한 검토가 필요할 수는 있겠으나 완치되어 격리가 해제될 때까지 무증상 상태였다. 이들 4명은 본인이 주관적으로 인지하는 증상의 발생이 없었을 뿐만 아니라 검사나 임상 경과 중 특이점도 발견할 수 없었다.

코로나바이러스감염증-19는 신종 감염병으로 무증상 감염과 관련되어 발표된 연구결과가 많지 않다. 최근 발표된 서울시 구로콜센터 집단발생에서 무증상감염률이 8.2%였던데 비해[6] 본 조사결과는 부산시 전체 확진 환자 98명 중 무증상자가 4명(4.0%)으로 낮게 나타났지만 무증상 감염이 가능하다는 동일한 결과를 보여 주었다.

무증상 확진 환자의 밀접 접촉자 조사 결과 1명(1.0%)의 추가 환자(K)가 발생하였으며 K는 무증상 확진 환자였던 가족 E로부터 감염되었을 것으로 보인다. 위에서 언급한 서울시 구로콜센터 집단발생 분석은 무증상기 전파여부에 대해 무증상자 16명의 접촉자 조사에서 무증상기 전파의 증거는 찾을 수 없었다고[7] 하였으나 본 연구에서는 1건의 무증상 전파 사례가 확인되었다.

밀접 접촉자였다가 확진된 K가 가족 중 먼저 확진된 E보다 증상발생이 선행하였으므로 두 사람의 감염 선후관계를 확인하기 위한 다각도의 조사를 시행한 결과 K는 가족인 E와의 접촉을 제외하고는 해외여행력, 코로나바이러스감염증-19 발생 장소 방문 및 다른 확진환자 접촉력 등 뚜렷한 감염원을 찾을 수 없었던 것에 비해 E는 감염원이 뚜렷하였으므로 E가 K에게 전파하였을 것으로 보는 것이 타당하다. K의 감염원을 확인하기 위해 2020.2.1. - 2.26.까지의 DUR 및 위치정보를 확인한 결과 동 기간 중 부산○○교회(K가 평소에 다니던 교회) 방문력과 서울방문(2.12-2.15.)이 확인되었다. 그러나 부산○○교회는 K 이외에 다른 환자 발생이 없었고, 위치정보를 통해 확인된 동선에서 서울방문 시 K는 김포공항에서 S병원으로 이동한 후 병원에만 머물렀으며 다른 지역 이동이 없었음이 확인되어 서울방문과 관련한 감염 가능성을 특기할 만한 상황은 발견할 수 없었다. 그러나 E는 부산 내 집단발생이 있었던 교회 수련회에 참석했다는 확실한 감염원 노출 경로를 확인할 수 있었다. 또한 E가 수련회에 참석하고 돌아온 시기는 방학기간으로 심층면담에서 본인들도 E가 집에 머물렀던 시간이 길었다고 진술하였다. 따라서 이 기간에 밀접한 노출이 지속적으로 이루어졌을 것으로 추정할 수 있겠다. 이런 상황들을 감안할 때 결국 증상발현은 K가 빨랐을지라도 E가 무증상기 동안 K에게 코로나바이러스감염증-19를 전파했을 것이라고 해석하는 것에 무리가 없다. 이런 결과는 독일에서의 직장동료 간 무증상기 전파를 보고한 선행 연구와[8] 맥락을 같이 한다.

WHO[9]와 중국[10]은 무증상 전파를 고려해서 확진 환자의 증상 발생 2일 전부터 접촉자 조사를 시행하도록 하고 있으나 한국은 「코로나바이러스감염증-19 대응지침 7-3판(지자체용)」[6]에서 증상발생 1일 전부터 접촉자 조사를 하도록 되어 있다. 코로나바이러스가 감염 초기에 전파력이 강하다는 것은 알려진 사실이므로 이에 대응하고 전파를 차단하기 위해서는 한국도 접촉자 조사범위를 확대하는 등 무증상 전파를 고려한 전파가능기간의 재검토가 요구된다.

**결론 Conclusion**

조사결과 부산시에 신고된 98명의 확진 환자 중 총 4명(4.0%)의 무증상 감염 환자를 발견할 수 있었다. 그 중 2명(2.0%)은 입원 시부터 치료제가 투여되어 증상발생을 억제했을 가능성에 대한 검토가 필요하다고 하더라도 적어도 다른 2명(2.0%)은 격리 해제일까지 무증상 상태였으므로 무증상 감염은 가능하다는 것을 확인하였다.

무증상기에 전파가 가능한가에 대해서는 환자의 무증상기에 밀접 접촉한 가족 중 1명의 추가 환자 발생이 확인되어 무증상기 전파 역시 가능하다는 것을 확인할 수 있었다. 그러나 본 조사는 10명의 적은 수를 대상으로 하였으므로 향후 추가 연구가 필요할 것이며, 아울러 무증상 감염률에 대한 보다 명확하고 과학적인 결과를 얻기 위해서는 면역학적 검사를 통한 보강된 조사가 필요할 것으로 사료된다.

**감사의 글 Acknowledgements**

부산광역시와 16개 구군 보건소, 환자치료에 힘쓰시는 의료진들을 비롯해서 코로나바이러스감염증-19 위기대응을 위해 곳곳에서 최선을 다하고 계신 모든 분들께 깊은 감사를 드립니다.

**이해 상충 Conflict of Interest**

The author has no conflicts of interest to declare for this study.

**참고 문헌 References**

1. World Health Organization. The First Few X cases and contacts (FFX) investigation protocol for coronavirus disease 2019 (COVID-19).[cited 2020 Jan 29]. Available from: <https://www.who.int/publications-detail/the-first-few-x-(ffx)-cases-and-contact-investigation-protocol-for-2019-novel-coronavirus-(2019-ncov)-infection>

2. Bai Y, Yao L, Wei T, et al. Presumed Asymptomatic Carrier Transmission of COVID-19. JAMA. Published online February 21, 2020. doi:10.1001/jama.2020.2565

3. Korean Ministry of Health Welfare. IMS meeting to respond to Novel Coronavirus is presided over by the Prime Minister[cited 2020 Feb 2]. Available from: <http://www.mohw.go.kr/react/al/sal0301vw.jsp?PAR_MENU_ID=04&MENU_ID=0403&page=1&CONT_SEQ=352649>

4. SARS-CoV-2 Viral Load in Upper Respiratory Specimens of Infected Patients. N Engl J Med. Mar 19, 2020;382:1177-1179, doi:10.1056/NEJMc2001737

5. Korea Centers for Disease Control and Prevention. COVID-19 guidelines (public health units, 7-4th edition), 34-35[cited 2020 Apr 2] Available from: https://www.cdc.go.kr/board/board.es?mid=a20507020000&bid=0019 (Korean).

6. Korean Ministry of Health Welfare, Korea Centers for Disease Control & Prevention. COVID-19 guidelines(public health units, 7-3th edition)[cited 2020 Mar 15]. Available from: <https://www.cdc.go.kr/board/board.es?mid=a20507020000&bid=0019>.

7. Korean Ministry of Health Welfare, Korea Centers for Disease Control & Prevention. The updates on COVID-19 in Korea as of 25 March. [cited 2020 Mar 25]. Available from: <https://www.cdc.go.kr/board/board.es?mid=a20501000000&bid=0015&list_no=366637&act=view>.

## 8. Camilla Rothe, Mirjam Schunk, Peter Sothmann et al. Transmission of 2019-nCoV Infection from an Asymptomatic Contact in Germany. N Eng J Med 2020;382:970-971

## 9. World Health Organization. Global surveillance for COVID-19 caused by human infection with COVID-19 virus: interim guidance, 20 March 2020[cited 2020 Mar 20]. Available from: [**https://apps.who.int/iris/handle/10665/331506**](https://apps.who.int/iris/handle/10665/331506).

## 10. National Health Commission of the People’s Republic of China. Investigation and manage- ment guidelines for close contacts of new cases of coronavirus pneumonia(6th edition) [cited 2020 Mar 7]. Available from: [**http://www.nhc.gov.cn/jkj/s3577/202003/4856d5b0458141fa9f376853224d41d7.shtml**](http://www.nhc.gov.cn/jkj/s3577/202003/4856d5b0458141fa9f376853224d41d7.shtml).
